# Sudden Sensorineural Hearing Loss Associated with Nutritional Anemia: A Nested Case–Control Study Using a National Health Screening Cohort

**DOI:** 10.3390/ijerph17186478

**Published:** 2020-09-05

**Authors:** So Young Kim, Jee Hye Wee, Chanyang Min, Dae-Myoung Yoo, Hyo Geun Choi

**Affiliations:** 1 Department of Otorhinolaryngology-Head & Neck Surgery, CHA Bundang Medical Center, CHA University, Seongnam 13496, Korea; sossi81@hanmail.net; 2 Department of Otorhinolaryngology-Head & Neck Surgery, Hallym University College of Medicine, 22, Gwanpyeong-ro 170beon-gil, Dongan-gu, Anyang-si, Gyeonggi-do 14068, Korea; weejh07@hanmail.net; 3 Hallym Data Science Laboratory, Hallym University College of Medicine, Anyang 14068, Korea; joicemin@naver.com (C.M.); ydm1285@naver.com (D.-M.Y.); 4 Graduate School of Public Health, Seoul National University, Seoul 01811, Korea

**Keywords:** hearing loss, sudden, anemia, cohort studies, case–control studies, hemoglobin

## Abstract

Previous studies have suggested an association of anemia with hearing loss. The aim of this study was to investigate the association of nutritional anemia with sudden sensorineural hearing loss (SSNHL), as previous studies in this aspect are lacking. We analyzed data from the Korean National Health Insurance Service-Health Screening Cohort 2002–2015. Patients with SSNHL (*n* = 9393) were paired with 37,572 age-, sex-, income-, and region of residence-matched controls. Both groups were assessed for a history of nutritional anemia. Conditional logistic regression analyses were performed to calculate the odds ratios (ORs) (95% confidence interval, CI) for a previous diagnosis of nutritional anemia and for the hemoglobin level in patients with SSNHL. Subgroup analyses were conducted for age and sex. Age, sex, income, and region of residence were stratified. Obesity, smoking, drinking alcohol, systolic/diastolic blood pressure, fasting blood glucose, total cholesterol, and the Charlson Comorbidity Index were considered in the regression models. Nutritional anemia was present in 4.8% (449/9393) of patients with SSNHL and 4.0% (1494/37,572) of controls (*p* < 0.001). The SSNHL group demonstrated 1.20-fold higher odds for nutritional anemia (95% CI = 1.08–1.34, *p* = 0.001). Hemoglobin levels were not associated with SSNHL. In subgroups <60 years old, there was a consistent positive association of nutritional anemia with SSNHL (adjusted OR = 1.55, 95% CI = 1.11–2.15, *p* = 0.010 for men <60 years old, and adjusted OR = 1.22, 95% CI = 1.02–1.45, *p* = 0.028 for women <60 years old). Nutritional anemia, but not hemoglobin level, was associated with an increased risk of SSNHL.

## 1. Introduction

Anemia is defined as a reduced red blood cell count and is characterized by a reduction in hemoglobin, which forms an essential component of red blood cells [1]. Anemia is one of the most common blood disorders and is estimated to affect approximately one-third of the total population, with a prevalence as high as 32.93% in preschool-aged children worldwide [2,3]. The etiology of anemia is multifactorial and characterized by an excessive loss of erythrocytes relative to erythrocyte production, which can occur due to nutritional deficiency, inflammation, or genetic conditions [3]. Nutritional deficiencies such as iron and vitamin B12 in anemic patients have been reported to be related with sensory disorders including hyposmia [4], olfactory craving (desiderosmia) [5], and hypogeusia [6]. In addition, anemia results in insufficient oxygen supply to various organs, which can cause behavioral dysfunction and cognitive impairment [7]. Therefore, organs with a higher oxygen demand might be more susceptible to an anemic insult. In addition, anemia predisposes to infection and inflammation [8,9].

The cochlea is one of the organs vulnerable to ischemic injury due to the high metabolic demands of cochlear hair cells involved in mechanoelectric transduction to acoustic stimuli. In addition, the cochlea receives its blood supply solely via the labyrinthine artery and has scanty collateral vasculature. Thus, oxidative stress and inflammation are the main pathophysiologic mechanisms for noise-induced, ototoxic drug-induced, and age-related hearing loss [10,11,12]. Sudden sensorineural hearing loss (SSNHL) is defined as a sudden onset (within three days) sensorineural hearing impairment in three continuous frequencies for ≥30 dB HL [13]. SSNHL is estimated to affect approximately 5–27 per 100,000 people annually [14]. The majority of cases of SSNHL (about 90%) are idiopathic. Its pathophysiologic causes are multifactorial and include viral infections, autoimmune disorders, and vascular abnormalities [14,15,16]. Several previous studies described the incidence of SSNHL in vascular disorders such as leukemia, sickle-cell anemia, and iron-deficiency anemia [17,18,19]. However, most of these studies were based on case series. A retrospective cohort study reported higher odds for iron-deficiency anemia in SSNHL [19], while the control group in this study was not matched for socioeconomic factors. We believe that selecting a control group matched for socioeconomic and demographic factors is essential, and this can be achieved by using health claims data obtained from clinical visits. In addition, to the best of our knowledge, no previous study has investigated the association of serum hemoglobin levels with SSNHL.

We hypothesized that nutritional anemia might be related to SSNHL. Because anemia has vascular, inflammatory, and metabolic effects, the association of nutritional anemia with SSNHL may not be solely dependent on the serum hemoglobin level. To test this hypothesis, we used a matched control group to analyze the association of nutritional anemia with SSNHL. We also explored the impact of hemoglobin status on SSNHL.

## 2. Materials and Methods

### 2.1. Study Population

The ethics committee of Hallym University (2019-10-023) approved this study. The requirement for written informed consent was waived by the Institutional Review Board. All analyses adhered to the guidelines and regulations of the ethics committee of Hallym University. A detailed description of The Korean National Health Insurance Service-Health Screening Cohort data has been previously published [20].

### 2.2. Participant Selection

**Figure 1:**
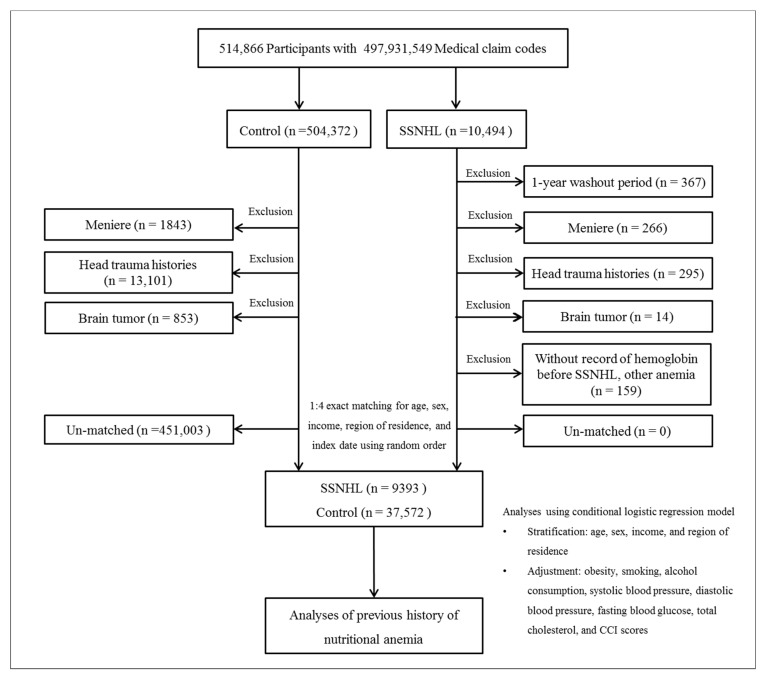
A schematic illustration of the participant selection process that was used in the present study. Of a total of 514,866 participants, 9393 of SSNHL patients were 1:4 matched with 37,572 control participants for age, sex, income, and region of residence.

We identified 514,866 participants after reviewing 615,488,428 medical claim codes from 2002 to 2015. Of these, 10,494 participants were assigned to the SSNHL group. The control group included participants without a diagnosis of SSNHL (*n* = 504,372). To select SSNHL patients who received a first-time diagnosis of SSNHL during the study period, those diagnosed in 2002 were excluded (washout period, *n* = 367). Participants were excluded if they met any of the following criteria: (1) treatment for Meniere’s disease (ICD-10 code: H810) ≥2 times and underwent audiometric examination (claim code: E6931–E6937, F6341–F6348) (*n* = 266 for SSNHL, *n* = 1843 for control); (2) treatment for traumatic head injury (ICD-10 codes: S00 to S09, diagnosed by neurologists, neurosurgeons, or emergency medicine doctors) and underwent head and neck computed tomography scan (claim codes: HA401–HA416, HA441–HA443, HA451–HA453, HA461–HA463, or HA471-HA473) (*n* = 295 for SSNHL, *n* = 13,101 for control); (3) treatment for brain tumor (ICD-10 codes: C70 to C72) ≥2 times (*n* = 14 for SSNHL, *n* = 853 for control); (4) missing data for hemoglobin level prior to treatment of SSNHL; and (5) diagnosis of hemolytic anemia (D55–D59), and aplastic and other anemias (D60–D64) (*n* = 159 for SSNHL). Excluded patients were not matched with the control group. Controls were matched to participants with SSNHL in terms of age, sex, income, and region of residence (1:4 matching). To minimize selection bias, the control participants were selected using random number generation. The index date of each SSNHL patient was set as the time of treatment of SSNHL. The index date of control participants was set as the index date of their matched SSNHL patients. Therefore, the index date was the same for each matched SSNHL and control participant. During the matching procedure, 451,003 control participants were excluded. The final analysis included 9393 SSNHL patients matched at a 1:4 ratio with 37,572 control participants (Figure 1).

### 2.3. Definition of Nutritional Anemia (Exposure)

Nutritional anemia was defined based on the presence of ICD-10 codes D50–D53 (nutritional anemias). To improve the accuracy of the diagnosis, we only selected participants who visited hospitals or clinics ≥2 times with a diagnosis of nutritional anemia.

### 2.4. Hemoglobin Concentration (Exposure)

The most recently available hemoglobin level prior to SSNHL diagnosis (index date) was used in the analysis.

### 2.5. Definition of Sudden Sensorineural Hearing Loss (Outcome)

Sudden sensorineural hearing loss (SSNHL) was defined using ICD-10 code H912 (sudden sensorineural hearing loss). We only included participants who underwent audiometric examination (claim code: E6931–E6937, F6341–F6348) and who were treated with steroids.

### 2.6. Covariates

Age groups were categorized using five-year intervals (40–44, 45–49, 50–54, 55–59, 60–64, 65–69, 70–74, 75–79, 80–84, and ≥85). Income groups were divided into five classes (class 1 (lowest income)–5 (highest income)). The region of residence was grouped into urban and rural areas [21]. Tobacco smoking, alcohol consumption, and obesity defined according body mass index (BMI, kg/m^2^) were included [22]. The Charlson Comorbidity Index (CCI) was included [23,24].

### 2.7. Statistical Analyses

The general characteristics of the SSNHL and control groups were compared. The paired sample *t*-test was used to compare continuous variables, and the chi-square test was used to compare categorical variables.

To analyze the odds ratios (ORs) (95% confidence intervals, CI), conditional logistic regression models for SSNHL with hemoglobin and nutritional anemia were calculated. The crude model, model 1 (adjusted for obesity, smoking, alcohol consumption, systolic blood pressure, diastolic blood pressure, fasting blood glucose, total cholesterol, and CCI scores), and model 2 (additionally adjusted for hemoglobin and nutritional anemia) were calculated. The analyses were stratified for age, sex, income, and region of residence.

For the subgroup analyses, we divided participants by age and sex (<60 years old and ≥60 years old; men and women) and analyzed the crude model, model 1, and model 2. We performed additional subgroup analyses by dividing participants by obesity (BMI < 23 and BMI ≥ 23), smoking (non-smoker and smoker), alcohol consumption (<1 time a week and ≥1 time a week), blood pressure (normal and hypertension), fasting blood glucose (<100 mg/dL and ≥100 mg/dL), and total cholesterol (<200 mg/dL and ≥200 mg/dL). Unconditional logistic regression models for osteoporosis in hemoglobin and nutritional anemia were calculated to analyze the ORs (95% CIs). The crude model, model 1 (adjusted for obesity, smoking, alcohol consumption, systolic blood pressure, diastolic blood pressure, fasting blood glucose, total cholesterol, and CCI scores), and model 2 (additionally adjusted for hemoglobin and nutritional anemia) were calculated.

Two-tailed analyses were performed, and significance was defined as *p* values less than 0.05. The SAS version 9.4 (SAS Institute Inc., Cary, NC, USA) was used for statistical analyses.

## 3. Results

A history of nutritional anemia was present in 4.8% (449/9393) of SSNHL patients and 4.0% (1494/37,572) of control participants (*p* < 0.001, Table 1). There were significant differences in obesity, smoking status, diastolic blood pressure, CCI score, and hemoglobin levels between the SSNHL and control groups (all *p* < 0.05). Alcohol consumption, systolic blood pressure, and fasting blood glucose did not differ between the SSNHL and control groups.

Nutritional anemia was positively associated with SSNHL (adjusted OR = 1.20, 95% CI = 1.08–1.34, *p* = 0.001, in Model 2, Table 2). However, the hemoglobin level was not related to SSNHL (adjusted OR = 1.02, 95% CI = 1.00–1.04, *p* = 0.106, in Model 2). In subgroup analysis, age <60 years showed higher odds for SSNHL in nutritional anemia patients (adjusted OR = 1.55, 95% CI = 1.11–2.15, *p* = 0.010, in Model 2 for men <60 years old; adjusted OR = 1.22, 95% CI = 1.02–1.45, *p* = 0.028, in Model 2 for women <60 years old, Table 2). There was no association of nutritional anemia with SSNHL in male and female patients ≥60 years old. Hemoglobin level was associated with higher odds for SSNHL only in women <60 years old (adjusted OR = 1.05, 95% CI = 1.00–1.09, in Model 2).

**Figure 2:**
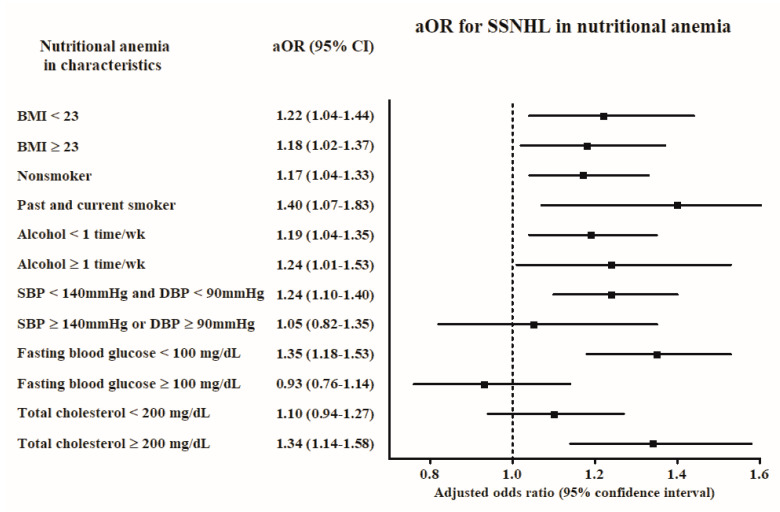
The odds ratios (95% confidence interval) of nutritional anemia for SSNHL according to obesity, smoking, alcohol consumption, blood pressure, fasting blood glucose, and total cholesterol.

**Figure 3:**
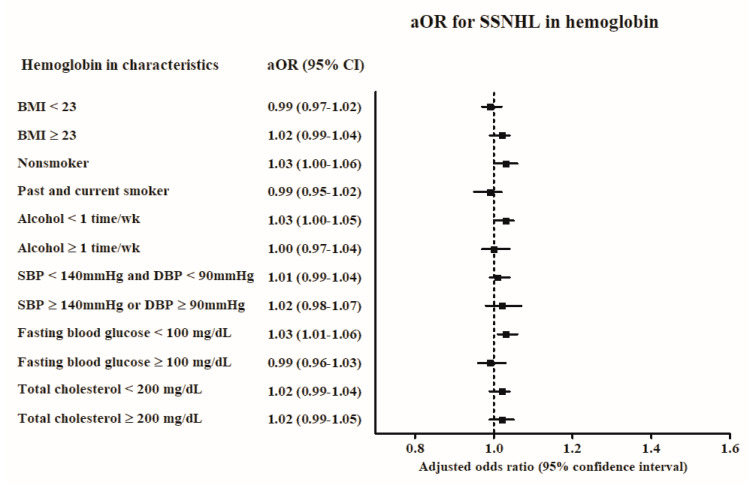
The odds ratios (95% confidence interval) of hemoglobin level for SSNHL according to obesity, smoking, alcohol consumption, blood pressure, fasting blood glucose, and total cholesterol.

The association of nutritional anemia with SSNHL was consistent in all subgroups adjusted for BMI, smoking status, and alcohol consumption (Table S1 and Figure 2). In addition, the normal blood pressure group (systolic blood pressure <140 mmHg and diastolic blood pressure <90 mmHg), the normal fasting blood glucose (<100 mg/dL) group, and the high total cholesterol (≥200 mg/dL) group demonstrated higher odds for SSNHL in patients with nutritional anemia. Hemoglobin level was associated with SSNHL in the non-smoker, alcohol consumption <1 time a week, and fasting blood glucose <100 mg/dL subgroups (Table S1, Figure 3).

## 4. Discussion

Nutritional anemia was positively related with SSNHL in patients aged ≥40 years old, and a significant association was only observed in patients aged <60 years old. However, the hemoglobin level was only associated with SSNHL in women <60 years old. This study used objective measures for BMI, blood pressure, fasting blood glucose, total cholesterol, and hemoglobin levels. These measures, in addition to obesity, smoking, alcohol consumption, and comorbidities assessed using CCI scores, were adjusted for when evaluating the relation between nutritional anemia or hemoglobin and SSNHL.

Previous studies have suggested an association between anemia and hearing loss [25,26,27]. In one study, the authors described 12 cases of SSNHL in 14 patients with leukemia or aplastic anemia [25]. A prospective cross-sectional study reported that 11.2% (10/89) of patients with sickle cell anemia had hearing loss [26]. A retrospective cohort study described 3.67-fold higher odds for SNHL in patients with iron deficiency anemia (95% CI = 1.60–7.30) [27]. Similarly, a case–control study reported 1.34-fold higher odds for SSNHL in patients with iron deficiency anemia (95% CI = 1.11–1.61) [19]. In the latter study, the association of iron deficiency anemia with SSNHL was strongest in patients aged ≤40 years old (adjusted OR = 1.91, 95% CI = 1.35–2.72) [27]. This age-specific association of anemia with SSNHL was concordant with that found our study. The higher prevalence of comorbidities and possible complex etiology of SSNHL in the elderly population might attenuate the association of anemia with SSNHL in this group. Our study adds to existing literature by also evaluating the association of hemoglobin level and cholesterol level with SSNHL in a large representative cohort population. In addition, we also adjusted for lifestyle factors including obesity, smoking, and alcohol.

The exact mechanism of nutritional anemia leading to SSNHL is unclear, but it is thought to include effects on the inner ear and auditory nerve mediated via pathophysiologic responses including hemorrhage, ischemia, ionic imbalance, and neurotrophic effects. For example, one theory proposes that SSNHL occurs due to ischemia of the perilymphatic or endolymphatic spaces in anemic patients. The cochlea is an organ with a high energy demand and end arterial blood supply that renders it vulnerable to ischemic insults [28]. Thus, the state of chronic ischemia in anemic patients could increase the risk of SSNHL [29]. In addition, thrombocytopenia in anemic patients might induce hemorrhage in the inner ear [30]. Several case reports have described SSNHL in leukemic patients caused by inner ear hemorrhage [30,31,32]. Moreover, anemic patients are more at risk of neutropenia, which can result in the infiltration of infective and inflammatory cells in the inner ear that cause SSNHL. Another theory is that biochemical and electrical imbalances in the inner ear of anemic patients could result in endolymphatic hydrops and SSNHL. A few previous studies have reported endolymphatic hydrops in patients with hematologic disorders such as anemia or leukemia [25,33].

Nutrient deficiency has also been suggested to cause defects in the auditory nervous system that may predispose to SSNHL. For instance, iron functions as a cofactor for neurotransmitter metabolism and DNA synthesis, and it is an essential component of the proteins regulating myelination and pruning of the nervous system [34,35]. Given its essential role in the normal function of the auditory nervous system, it is plausible that iron deficiency could adversely affect the auditory nervous system. This theory is supported by an experimental study in an iron-deficient rat model with multiple inner ear defects, including scanty number of spiral ganglion cells and degeneration of the stereocilia of the outer and inner hair cells [36]. In humans, a clinical trial in children demonstrated that, compared to controls, children who were treated for iron deficiency anemia showed delayed latencies of the auditory brainstem response [37]. The authors suggested that the effects of altered myelination of the auditory nervous system of children with iron deficiency anemia might be sustained [37]. Further, it has been suggested that the effects of anemia on the auditory nervous system may be long lasting despite iron supplementation and normalization of hemoglobin levels [38]. Indeed, our study found that nutritional anemia was positively associated with SSNHL, but there was no association between the hemoglobin level and SSNHL.

This study had several strengths. First, the study used a large representative cohort population, and the control group was matched for age, sex, income, and region of residence. As this cohort underwent a comprehensive health screening assessment that included blood tests for hemoglobin and total cholesterol levels, as well as measurement of BMI and blood pressure, we were able to analyze the effect of these objective measures in the association between SSNHL and nutritional anemia. The inclusion of laboratory data allowed us to evaluate the association of hemoglobin level with SSNHL.

This study also has certain limitations worth noting. First, there was a lack of information available on the medical claim data. Second, data were only available for patients who sought medical advice in clinics, and it is possible that subclinical or undiagnosed cases were missed. Third, data on the severity of nutritional anemia and SSNHL and the prognosis of affected patients were unavailable. Finally, although we adjusted for numerous potential confounders such as obesity, smoking, alcohol consumption, blood pressure, fasting blood glucose, total cholesterol, and CCI scores, there could have been other confounders that we did not adjust for, such as history of noise exposure, which could have influenced the results [39,40]. Future study with the participants with specified types of anemia and SSNHL will delineate the current questions. In addition, pre-clinical studies for the underlying pathophysiology for the association of nutritional anemia with SSNHL could pave the way for the prevention and treatment of SSNHL related with nutritional anemia. Although the current results could not determine the causality between nutritional anemia and SSNHL, the early diagnosis and management of anemia in SSNHL and vice versa might be beneficial to treatment of both diseases.

## 5. Conclusions

Nutritional anemia was associated with increased odds for SSNHL in this study. This association was consistent in the younger adult population in both male and female subgroups. On the other hand, the hemoglobin level was not related with SSNHL, which implied the chronic effects of nutritional anemia on SSNHL.

## Figures and Tables

**Table 1:** General characteristics of participants.

Characteristics		Total Participants		
		SSNHL	Control	*p*-Value
Age (years old, *n*, %)				1.000
	40–44	159 (1.7)	636 (1.7)	
	45–49	867 (9.2)	3468 (9.2)	
	50–54	1832 (19.5)	7328 (19.5)	
	55–59	1954 (20.8)	7816 (20.8)	
	60–64	1616 (17.2)	6464 (17.2)	
	65–69	1279 (13.6)	5116 (13.6)	
	70–74	952 (10.1)	3808 (10.1)	
	75–79	522 (5.6)	2088 (5.6)	
	80–84	182 (1.9)	728 (1.9)	
	85+	30 (0.3)	120 (0.3)	
Sex (*n*, %)				1.000
	Male	4857 (51.7)	19,428 (51.7)	
	Female	4536 (48.3)	18,144 (48.3)	
Income (*n*, %)				1.000
	1 (lowest)	1330 (14.2)	5320 (14.2)	
	2	1118 (11.9)	4472 (11.9)	
	3	1415 (15.1)	5660 (15.1)	
	4	1997 (21.3)	7988 (21.3)	
	5 (highest)	3533 (37.6)	14,132 (37.6)	
Region of residence (*n*, %)				1.000
	Urban	4134 (44.0)	16,536 (44.0)	
	Rural	5259 (56.0)	21,036 (56.0)	
Obesity ‡ (*n*, %)				0.001 *
	Underweight	166 (1.8)	866 (2.3)	
	Normal	3209 (34.2)	12,979 (34.5)	
	Overweight	2680 (28.5)	10,595 (28.2)	
	Obese I	3096 (33.0)	11,993 (31.9)	
	Obese II	242 (2.6)	1139 (3.0)	
Smoking status (*n*, %)				
	Nonsmoker	6770 (72.1)	26,173 (69.7)	<0.001 *
	Past smoker	1335 (14.2)	5013 (13.3)	
	Current smoker	1288 (13.7)	6386 (17.0)	
Alcohol consumption (*n*, %)				
	<1 time a week	6197 (66.0)	24,420 (65.0)	0.074
	≥1 time a week	3196 (34.0)	13,152 (35.0)	
Systolic blood pressure (*n*, %)				
	<120 mmHg	3022 (32.2)	11,767 (31.3)	0.114
	120–139 mmHg	4582 (48.8)	18,339 (48.8)	
	≥140 mmHg	1789 (19.1)	7466 (19.9)	
Diastolic blood pressure (*n*, %)				0.002 *
	<80 mmHg	4620 (49.2)	17,736 (47.2)	
	80–89 mmHg	3297 (35.1)	13,604 (36.2)	
	≥90 mmHg	1476 (15.7)	6232 (16.6)	
Fasting blood glucose (*n*, %)				0.759
	<100 mg/dL	5832 (62.1)	23,438 (62.4)	
	100–125 mg/dL	2688 (28.6)	10,609 (28.2)	
	≥126 mg/dL	873 (9.3)	3525 (9.4)	
Total cholesterol (*n*, %)				0.158
	<200 mg/dL	5042 (53.7)	19,863 (52.9)	
	200–239 mg/dL	3140 (33.4)	12,603 (33.5)	
	≥240 mg/dL	1211 (12.9)	5106 (13.6)	
CCI score (mean, SD)		0.56 (1.1)	0.41 (0.9)	<0.001 ^†^
Hemoglobin (mean, SD)		13.5 (1.4)	13.8 (1.5)	<0.001 ^†^
Nutritional anemia (*n*, %)		449 (4.8)	1494 (4.0)	<0.001 *

Abbreviations: CCI, Charlson Comorbidity Index calculated without cancer and metastatic cancer; SSNHL, sudden sensorineural hearing loss. * Chi-square test. Significance at *p* < 0.05. ^†^ Paired-sample *t*-test. Significance at *p* < 0.05. ^‡^ Obesity (BMI, body mass index, kg/m^2^) was categorized as <18.5 (underweight), ≥18.5 to <23 (normal), ≥23 to <25 (overweight), ≥25 to <30 (obese I), and ≥30 (obese II).

**Table 2:** Crude and adjusted odds ratios (95% confidence interval) for SSNHL in hemoglobin and nutritional anemia according to age and sex.

Characteristics		Odds Ratios for SSNHL					
		Crude ^†^	*p*-Value	Model 1 ^†,‡^	*p*-Value	Model 2 ^†,§^	*p*-Value
Total participants (*n* = 45,965)							
	Hemoglobin	1.00 (0.98–1.02)	0.871	1.01 (0.99–1.03)	0.235	1.02 (1.00–1.04)	0.106
	Nutritional anemia	1.22 (1.09–1.36)	<0.001 *	1.19 (1.06–1.32)	0.002 *	1.20 (1.08–1.34)	0.001 *
Age < 60 years old, men (*n* = 12,655)							
	Hemoglobin	0.96 (0.92–1.00)	0.034 *	0.97 (0.93–1.01)	0.154	0.98 (0.94–1.02)	0.286
	Nutritional anemia	1.70 (1.23–2.35)	0.001 *	1.59 (1.14–2.20)	0.006 *	1.55 (1.11–2.15)	0.010 *
Age < 60 years old, women (*n* = 11,405)							
	Hemoglobin	1.03 (0.99–1.07)	0.163	1.04 (1.00–1.08)	0.085	1.05 (1.00–1.09)	0.039 *
	Nutritional anemia	1.20 (1.01–1.42)	0.042 *	1.18 (0.99–1.40)	0.062	1.22 (1.02–1.45)	0.028 *
Age ≥ 60 years old, men (*n* = 11,630)							
	Hemoglobin	1.00 (0.97–1.04)	0.981	1.01 (0.97–1.05)	0.688	1.01 (0.98–1.05)	0.510
	Nutritional anemia	1.28 (1.01–1.62)	0.042 *	1.24 (0.98–1.58)	0.077	1.26 (0.99–1.60)	0.065
Age ≥ 60 years old, women (*n* = 11,275)							
	Hemoglobin	1.03 (0.98–1.07)	0.232	1.04 (0.99–1.08)	0.105	1.04 (0.99–1.09)	0.093
	Nutritional anemia	1.06 (0.86–1.30)	0.578	1.05 (0.85–1.29)	0.689	1.07 (0.87–1.32)	0.538

Abbreviation: CCI, Charlson Comorbidity Index; SSNHL, sudden sensorineural hearing loss. * Conditional logistic regression model, significance at *p* < 0.05. ^†^ Models stratified by age, sex, income, and region of residence. ^‡^ Model 1 was adjusted for obesity, smoking, alcohol consumption, systolic blood pressure, diastolic blood pressure, fasting blood glucose, total cholesterol, and CCI scores. ^§^ Model 2 was adjusted for model 2 with hemoglobin and nutritional anemia.

## Supplementary Materials

The following are available online at https://www.mdpi.com/1660-4601/17/18/6478/s1, Table S1: Subgroup analyses of crude and adjusted odds ratios (95% confidence interval) for SSNHL in hemoglobin and nutritional anemia according to obesity, smoking, alcohol consumption, blood pressure, fasting blood glucose, and total cholesterol.
