# Supplementary material for: Sudden Sensorineural Hearing Loss Associated with Nutritional Anemia: A Nested Case–Control Study Using a National Health Screening Cohort
Source: Int J Environ Res Public Health. 2020 Sep 5;17(18):6478. doi: 10.3390/ijerph17186478 (PMC7558085; doi:10.3390/ijerph17186478)
Supplement: Supplementary file 1 [file ijerph-17-06478-s001.pdf]

Table S1. Subgroup analyses of crude and adjusted odd ratios (95% confidence interval) for SSNHL in hemoglobin and nutritional anemia according to obesity, smoking, alcohol consumption, blood pressure, fasting blood glucose, and total cholesterol.

Characteristics	Odd Ratios for SSNHL					
	Crude	*p*-Value	Model 1 †	*p*-Value	Model 2 ‡	*p*-Value
BMI < 23 (*n* = 17,220)						
Hemoglobin	0.99 (0.97–1.02)	0.684	1.02 (0.98–1.05)	0.314	0.99 (0.97–1.02)	0.314
Nutritional anemia	1.24 (1.06–1.46)	<0.001 *	1.20 (1.02–1.41)	0.029 *	1.22 (1.04–1.44)	0.018 *
BMI ≥ 23 (*n* = 29,745)						
Hemoglobin	1.00 (0.98–1.02)	0.864	1.02 (0.99–1.04)	0.352	1.02 (0.99–1.04)	0.245
Nutritional anemia	1.20 (1.04–1.39)	0.016 *	1.16 (1.01–1.35)	0.043 *	1.18 (1.02–1.37)	0.031 *
Nonsmoker (*n* = 32,943)						
Hemoglobin	1.02 (1.01–1.04)	0.012 *	1.02 (1.00–1.05)	0.060	1.03 (1.00–1.06)	0.022 *
Nutritional anemia	1.15 (1.03–1.30)	0.018 *	1.15 (1.02–1.30)	0.022 *	1.17 (1.04–1.33)	0.010 *
Past smoker and current smoker (*n* = 14,022)						
Hemoglobin	0.99 (0.96–1.03)	0.734	0.98 (0.95–1.02)	0.249	0.99 (0.95–1.02)	0.410
Nutritional anemia	1.41 (1.09–1.83)	0.009 *	1.42 (1.09–1.85)	0.009 *	1.40 (1.07–1.83)	0.013 *
Alcohol consumption < 1 time a week (*n* = 30,617)						
Hemoglobin	1.01 (0.99–1.03)	0.216	1.02 (1.00–1.05)	0.087	1.03 (1.00–1.05)	0.039 *
Nutritional anemia	1.19 (1.05–1.35)	0.008 *	1.16 (1.02–1.32)	0.024 *	1.19 (1.04–1.35)	0.011 *
Alcohol consumption ≥ 1 time a week (*n* = 16,348)						
Hemoglobin	0.99 (0.97–1.02)	0.538	1.00 (0.96–1.03)	0.821	1.00 (0.97–1.04)	0.988
Nutritional anemia	1.26 (1.03–1.54)	0.025 *	1.24 (1.01–1.53)	0.036 *	1.24 (1.01–1.53)	0.040 *
Systolic blood pressure < 140 mmHg and diastolic blood pressure < 90 mmHg (*n* = 35,666)						
Hemoglobin	1.00 (0.99–1.02)	0.844	1.01 (0.99–1.03)	0.436	1.01 (0.99–1.04)	0.218
Nutritional anemia	1.25 (1.10–1.41)	<0.001 *	1.23 (1.08–1.38)	0.001 *	1.24 (1.10–1.40)	0.001 *
Systolic blood pressure ≥ 140 mmHg or diastolic blood pressure ≥ 90 mmHg (*n* = 11,299)						
Hemoglobin	1.00 (0.97–1.04)	0.845	1.02 (0.98–1.06)	0.300	1.02 (0.98–1.07)	0.277
Nutritional anemia	1.08 (0.85–1.38)	0.530	1.03 (0.81–1.32)	0.799	1.05 (0.82–1.35)	0.678
Fasting blood glucose < 100 mg/dL (*n* = 29,270)						
Hemoglobin	1.01 (1.00–1.03)	0.149	1.03 (1.00–1.05)	0.057	1.03 (1.01–1.06)	0.013 *

Nutritional anemia	1.32 (1.16–1.50)	<0.001 *	1.31 (1.15–1.50)	<0.001 *	1.35 (1.18–1.53)	<0.001 *
Fasting blood glucose ≥ 100 mg/dL (*n* = 17,695)						
Hemoglobin	0.98 (0.96–1.00)	0.088	1.00 (0.96–1.03)	0.747	0.99 (0.96–1.03)	0.674
Nutritional anemia	1.00 (0.82–1.22)	0.986	0.94 (0.76–1.15)	0.515	0.93 (0.76–1.14)	0.481
Total cholesterol < 200 mg/dL (*n* = 24,905)						
Hemoglobin	1.01 (0.99–1.03)	0.577	1.01 (0.99–1.04)	0.285	1.02 (0.99–1.04)	0.214
Nutritional anemia	1.10 (0.96–1.27)	0.184	1.08 (0.93–1.25)	0.310	1.10 (0.94–1.27)	0.230
Total cholesterol ≥ 200 mg/dL (*n* = 22,060)						
Hemoglobin	1.00 (0.97–1.02)	0.719	1.01 (0.98–1.04)	0.529	1.02 (0.99–1.05)	0.298
Nutritional anemia	1.37 (1.17–1.62)	<0.001 *	1.34 (1.14–1.58)	<0.001 *	1.34 (1.14–1.58)	<0.001 *

Abbreviation: CCI, Charlson Comorbidity Index; SSNHL, Sudden sensorineural hearing loss;* Logistic regression model, Significance at $p < 0.05$; † Model 1 was adjusted for obesity, smoking, alcohol consumption, systolic blood pressure, diastolic blood pressure, fasting blood glucose, total cholesterol, and CCI scores; ‡ Model 2 was adjusted for model 2 with hemoglobin and nutritional anemia.
